# Formulation, Characterization, and Evaluation of Eudragit-Coated Saxagliptin Nanoparticles Using 3 Factorial Design Modules

**DOI:** 10.3390/molecules27217510

**Published:** 2022-11-03

**Authors:** Yahya Alhamhoom, Gundawar Ravi, Riyaz Ali M. Osmani, Umme Hani, Gowrav M. Prakash

**Affiliations:** 1 Department of Pharmaceutics, College of Pharmacy, King Khalid University, Abha 62529, Saudi Arabia; 2 Department of Pharmaceutical Quality Assurance, Manipal College of Pharmaceutical Sciences, Manipal Academy of Higher Education, Manipal 576104, Karnataka, India; 3 Department of Pharmaceutics, JSS College of Pharmacy, JSS Academy of Higher Education and Research, Mysuru 570015, Karnataka, India

**Keywords:** type 2 diabetes mellitus, Saxagliptin, solid lipid nanoparticles, Quality by Design, bioavailability, stability

## Abstract

**Background and Introduction:** Saxagliptin is a hypoglycemic drug that acts as a dipeptidyl peptidase-4 (DPP-4) inhibitor and is preferably used in the treatment of Type 2 Diabetes Mellitus (T2DM). It is safe and tolerable; however, the major disadvantage associated with it is its low bioavailability. **Aim:** The present research aimed to enhance the bioavailability of the drug by enteric coating with a polymer that controls the rate of drug delivery, and it was prepared as Solid Lipid Nanoparticles (SLNs). **Methodology:** In the current study, various SLN formulations were developed using a central composite design (CCD) module using Design Expert-11 software. A modified solvent injection technique was used to prepare Saxagliptin nanoparticles coated with Eudragit RS100. The CCD was used to determine the independent variables and their effect on dependent variables at varied levels. Evaluation studies such as particle size analysis, Zeta potential, polydispersity index (PDI), drug loading, entrapment efficiency, in-vitro drug release studies, and in vivo pharmacokinetic studies were performed for the optimized SLN formulation. The reversed-phase HPLC method was developed and validated for the estimation of the pharmacokinetic parameters of the pure drug and prepared SLNs. **Results:** The effect of independent variables (A1: amount of lipid, A2: amount of polymer, A3: surfactant concentration, and A4: homogenization speed) on dependent variables (R1: particle size, and R2: entrapment efficiency) was established in great detail. Observed responses of the prepared and optimized Saxagliptin SLN were close to the predicted values by the CCD. The prepared SLNs depicted particle sizes in the range of 212–442 nm. The particle size analysis results showed that an increase in the lipid concentration led to an increase in particle size. The developed bioanalytical method was noted to be very specific and robust. The method accuracy varied from 99.16% to 101.95% for intraday, and 96.08% to 103.12% for inter day operation at low (5 mcg/mL), moderate (10 mcg/mL), and higher (15 mcg/mL) drug concentrations. The observed Zeta potential values for the prepared SLNs were in the range of −41.09 ± 0.11 to 30.86 ± 0.63 mV suggesting quite good stability of the SLNs without any aggregation. Moreover, the polydispersity indices were in the range of 0.26 ± 0.051 to 0.45 ± 0.017, indicative of uniformity of sizes among the prepared SLNs. In vivo study outcomes proved that Saxagliptin oral bioavailability significantly enhanced in male Albino Wistar Rats via SLN formulation and Eudragit RS100 coating approach. **Conclusions:** The developed and optimized Saxagliptin SLNs revealed enhanced Saxagliptin bioavailability in comparison to the native drug. Thus, this formulation strategy can be of great importance and can be implied as a promising approach to enhance the Saxagliptin bioavailability for facilitated T2DM therapy.

## 1. Introduction

Type 2 diabetes Mellitus (T2DM) is the most frequent type, accounting for around 90–95% of all diabetic cases. Metformin is the regular drug recommended by physicians for people suffering from T2DM [1]. Diabetes Mellitus (DM) is a chronic metabolic disorder that results due to decreased or very minor insulin secretion leading to increased sugar levels in the blood [2,3]. Day-by-day percentage of people being affected by this disorder is increasing which may be because of age factors and unhealthy lifestyles [4,5]. At present, ample research studies are working to find novel methods and treatments to manage diabetes. One among them that has become more popular in recent times is nanomedicine usage in managing diabetes [6].

**Figure 1:**
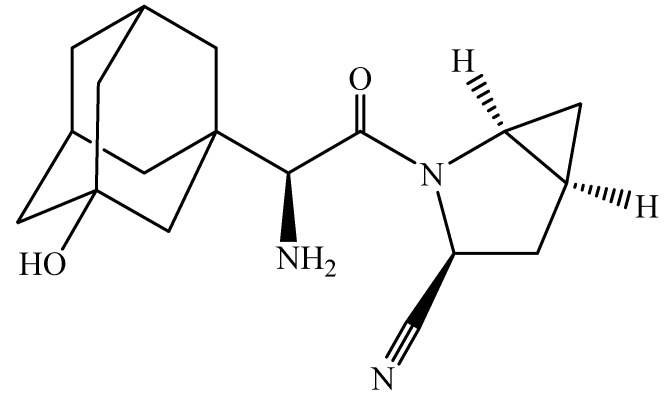
Chemical structure of Saxagliptin.

Saxagliptin [(S)-3-hydroxyadamantylglycine-L-*cis*-4,5-methanoprolinenitrile], approved by the U.S. Food and Drug Administration (FDA) is a dipeptidyl peptidase-4 (DPP-4) inhibitor used in the therapy of T2DM in adults [7]. DPP-4 inhibitors act by decreasing the deactivation of the incretins. Incretins stimulate the insulin secretion of pancreatic *β*-cells and inhibit the hepatic glucagon production of *α*-cells. They help in reducing blood sugar facilitating higher sugar consumption by the body [8,9]. DPP-4 is a peptidase associated with membranes and found in most tissues, lymphocytes, and plasma. The major two mechanisms of action of DPP-4 are (a) an enzymatic function and (b) DPP-4 binds with adenosine deaminase, which conveys intracellular signals through dimerization upon activation [10,11]. Saxagliptin is one of the DPP-4 inhibitors which is safer and more tolerable [12]. It neither increases the risk of cardiovascular events nor decreases renal function [13]. Hepatic first-pass metabolism is the main disadvantage associated with this drug, followed by a high elimination rate and low bioavailability (67%) [14]. The chemical structure of Saxagliptin is shown in Figure 1 [15].

Saxagliptin is currently available in a 2.5 mg dose with poor membrane permeability that ultimately results in lesser oral bioavailability of up to 50%. Its solubility in the water further leads to a short elimination half-life (4–6 h), hence demanding multiple daily dosing. Moreover, the uncontrolled release from conventional tablets and higher systemic concentrations of Saxagliptin leads to gastrointestinal disturbances such as diarrhea, stomach pain, vomiting, bloating, and nausea. Furthermore, it is also a substrate for the P-glycoprotein-driven efflux that also contributes to the rapid removal of the drug [16]. Thus, a controlled release dosage form may prove to be a good choice to solve the aforementioned drug problem and may enhance the patient’s compliance.

Solid lipid nanoparticles (SLNs) owing to their low toxicity, excellent biodegradability, and high physical stability, are gaining more consideration currently in drug delivery and therapeutics. Another boon with them is that they can carry both hydrophilic and hydrophobic drugs and renders sustained delivery of these drugs. Apart from being economical and easy to formulate, they can play a major role in increasing the oral bioavailability of drugs that are poorly water-soluble [17]. Oral administration of these nanoparticles enhances drug lymphatic transport and bypasses hepatic first-pass metabolism [18]. In the past, in vivo pharmacokinetic studies on Albino Wistar rats were performed to determine the oral bioavailability and biodistribution. Our extensive literature review revealed that there are limited research attempts made and studies undertaken to aim primarily at enhancing the oral bioavailability of Saxagliptin [19,20]. Preparation of Saxagliptin SLNs using Eudragit RS100 as a coating material has not been reported yet in any research study to date. Thus, in the present research work, we aimed and attempted to develop a formulation that would increase the oral bioavailability of Saxagliptin.

The prime objective of current work was to design and develop Saxagliptin SLNs coated with Eudragit RS100 with an intent to reduce the dosing frequency and to enhance the oral bioavailability. Modified solvent injection technique was used to prepare Saxagliptin SLNs implying Quality by Design (QbD) approach using the Central Composite Design (CCD) to determine the independent variables and their effect on fixed dependent variables. Evaluation studies such as particle size analysis, Zeta potential, polydispersity index (PDI), drug loading, entrapment efficiency, in vitro drug release studies, and in vivo pharmacokinetic studies were performed for the optimized SLN formulation.

## 2. Results and Discussion

### 2.1. Drug Solubility Studies

The solubility of Saxagliptin in glyceryl monostearate and stearic acid was evaluated for the appropriate lipid selection. Obtained solubility results in stearic acid and glyceryl monosterate were 0.58 ± 0.65 mg/mL and 0.42 ± 0.09 mg/mL, respectively. Stearic acid depicted comparatively more drug solubilization potential; hence, it was selected for Saxagliptin SLNs preparation.

### 2.2. Determination of Particle Size, Zeta Potential and Polydispersity Index

Considering the hydrophilic nature of Saxagliptin, a modified solvent injection technique was adopted for the preparation of Saxagliptin SLNs. Parameters such as lipid, surfactant and drug concentrations that affect the physicochemical properties of SLNs play a very crucial role in determining the particle size of SLNs. The emulsifier (Poloxamer188) and stabilizer (polyvinyl alcohol) used during the preparation prevent the aggregation and/or agglomeration and improve SLNs stability. Prepared SLNs were lastly coated with Eudragit RS100 for prolonging the release and thereby enhancing the oral bioavailability of the drug.

A particle size analyzer and SEM were used to analyze and study the prepared Saxagliptin SLNs particle size and shape. The particle size analysis results showed that an increase in the lipid concentration led to an increase in particle size, i.e., direct proportionality; whereas, surfactant concentration and particle size were inversely proportional to each other. This may be because at higher concentrations, lipid tends to aggregate forming larger particles. Moreover, a rise in the lipid amount provides extra space to the drug for becoming entrapped, subsequently, resulting in decreased particle surface area and increased particle size [21,22]. On the other hand, with an increased surfactant concentration reverse phenomenon occurs that results in reduced particle size.

The observed Zeta potential values for the prepared SLNs were in the range of −41.09 ± 0.11 to 30.86 ± 0.63 mV suggesting quite good stability of the SLNs without any aggregation. Moreover, the polydispersity indices were in the range of 0.26 ± 0.051 to 0.45 ± 0.017, indicative of uniformity of sizes among the prepared SLNs.

### 2.3. Determination of DL and EE

The DL of the prepared SLN batches was noted in the range of 16.99 ± 0.8% to 25 ± 1.3%. The noted DL values were satisfactory and established the relation that with an increase in lipid concentration (from 5% to 15%) the DL gradually decreases. On the other hand, EE noted for the SLN batches was in the range of 49.96 ± 0.9% to 69.21 ± 1.8%, and indicated that with an increase in lipid concentration, EE also increases. Furthermore, both the DL and EE depicted direct proportionality relation with the surfactant concentration. This may be due to the availability or adsorption of free drug onto the surface of SLNs [23,24].

### 2.4. In Vitro Diffusion Study

**Figure 2:**
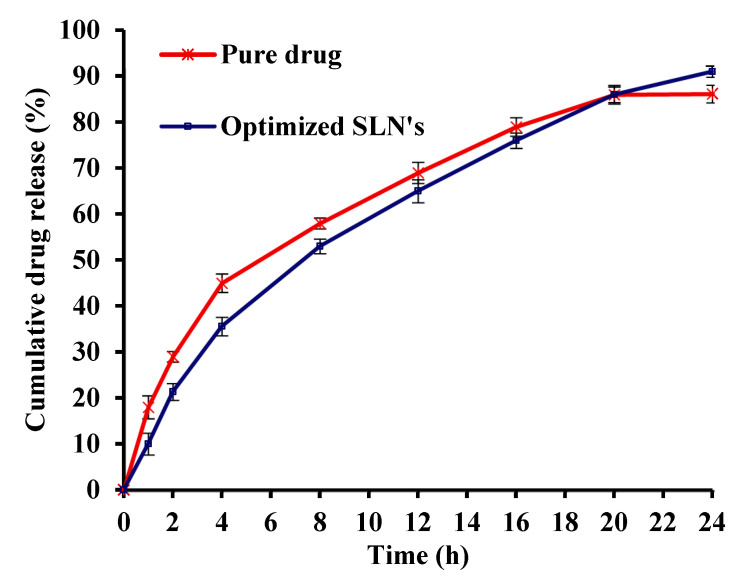
In vitro diffusion study release profiles of pure drug and optimized Saxagliptin SLNs.

An in vitro diffusion study was carried out for pure drug and optimized SLNs in dissolution media of pH 7.4 (Figure 2). It was found that due to Eudragit RS100 coating and increased polymer concentration, the release of Saxagliptin from the optimized SLNs decreased. This can be attributed to the effect of buffer pH on the swelling of Eudragit RS100 (coating material) that subsequently influences the drug release from SLNs. Eudragit Rs100 is a pH-dependent methacrylic acid copolymer which has less permeability for the dissolution medium; hence, increasing the amount of polymer hindered the penetration of the dissolution medium into the SLNs and the subsequent drug dissolution and diffusion [25,26]. Upon fitting the release kinetics data to diverse mathematical models, the best fit kinetic model for SLNs was Peppas’ model with a regression coefficient value (R^2^) of 0.991.

### 2.5. Experimental Design

CCD is used to reduce variability in any preparation technique and to enhance or improve the accuracy of process parameter optimization that ultimately boosts product yield and reduces drug-excipients wastage. The data from all the formulations were analyzed in order to come up with software suggested optimized formulation. ANOVA was applied to find factors that had a significant impact on the predetermined responses. The outcomes of experimental design-based optimization revealed that selected independent variables had significant impact on the dependent variable at all the three levels (low- −1, medium- 0, high- +1). Details of the applied design are quoted in Table 1.

#### Response 1: Particle Size

**Figure 3:**
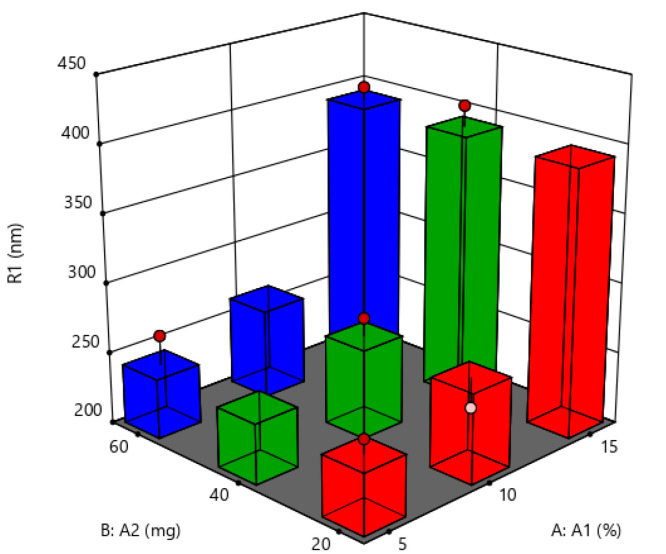
Three-dimensional response surface plot for the dependent variable particle size (R1).

To determine the physical stability of the formulations, particle sizes and their distribution were considered and noted as the crucial evaluation parameters. Impact of independent variables, i.e., lipid amount (A1), polymer amount (A2), concentration of surfactant (A3), and homogenization speed (A4) on the dependent variable particle size (R1) was studied and noted results are tabulated in Table 1. In the case of response 1 (R1: particle size) the 2FI model was found to be significant with F-value and *p*-value of 5.62 and 0.0311, respectively. Independent variables or factors A: amount of lipid and B: amount of polymer depicted quite significant influence on particle size, whereas factors C: surfactant concentration and D: homogenization speed showed a negative effect on the response particle size. At increased concentrations of lipid and polymer, the probability of particle aggregation is more likely, reducing emulsifying efficacy. Gaps between individual particles increase with greater surfactant concentrations, which prevents particle agglomeration and reduces particle size. At lower homogenization speeds, again there is more prevalence of SLNs aggregation, resulting in greater particle sizes. This can also be correlated to the role of homogenization speed in particle size reduction for the prepared SLNs by means of converting coarse particles to nanoparticles. The noted effects of factors on the particle size of SLNs are presented in the form of 3D response surface plot in Figure 3.

### 2.6. Particle Size ANOVA for 2FI Model

The model F-value of 5.62 implies that the model is significant and there is only a 3.11% chance that an F-value this large can occur due to noise. The model *p*-value was less than 0.0500 which indicated significance in model terms. Model reduction may enhance the model if it has a large number of insignificant model terms (except those necessary to maintain the hierarchy). Particle size ANOVA Response for 2FI model for the prepared Saxagliptin SLNs is provided in Table 2.

#### Response 2: Entrapment Efficiency

**Figure 4:**
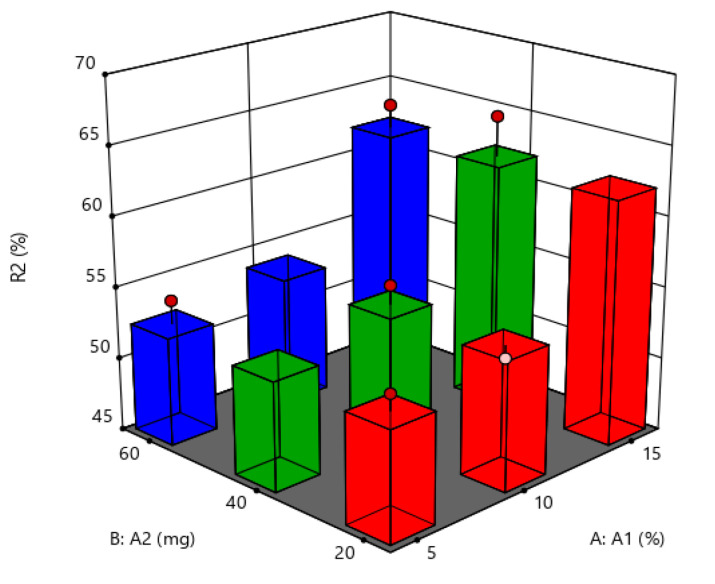
Three-dimensional response surface plot for the dependent variable entrapment efficiency (R2).

For the dependent variable EE, the F-value and *p*-value obtained were 5.68 and 0.0304, respectively. These values suggested that applied 2FI model was significant for the specified response. All terms were significant with *p*-value ˂ 0.0001. Predicted R^2^ was found to be 0.9986, and adjusted R^2^ was 0.9953, indicating minor variations existed in experimental model. Factors amount of lipid (A) and amount of polymer (B) showed positive coefficients stating direct proportionality of these factors with the response EE. Whereas factor surfactant concentration (C) exhibited a negative coefficient suggesting that it has indirect proportional relation with the response EE. The noted effects of factors on the EE of SLNs are presented in the form of 3D response surface plot in Figure 4.

### 2.7. EE ANOVA for 2FI Model

The model F-value of 5.68 suggests that the model is significant and there is only a 3.04% chance for occurrence of larger F-value such as this due to the noise. Model *p*-values less than 0.0500 indicate that significant model terms are present. Here A is a model term that is significant. Model terms are considered not significant if values are greater than 0.1000. EE ANOVA Response for 2FI model for the prepared Saxagliptin SLNs is provided in Table 3.

### 2.8. Desirability

**Figure 5:**
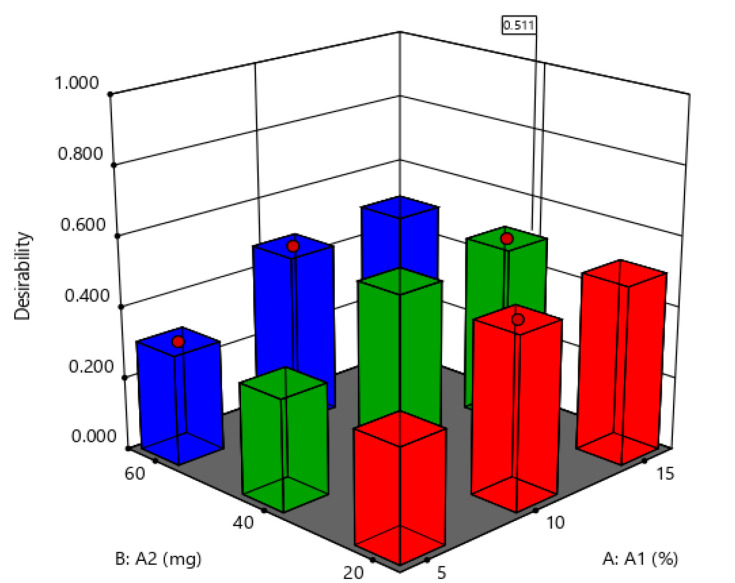
Three-dimensional surface plot of desirability for the optimized SLN formulation design.

For optimization of SLNs, the formulation with the highest desirability was selected. For any provided response, the desirability value ranges from 0 to 1. The ideal case is represented by value 1, and 0 indicates one or more responses which fall outside the desirable limits [27]. For our optimized SLN formulation the noted desirability value was 0.511 (Figure 5), which suggests that the implemented design was significant, optimized accurately, and was robust in terms of all coefficients.

### 2.9. FTIR Analysis

**Figure 6:**
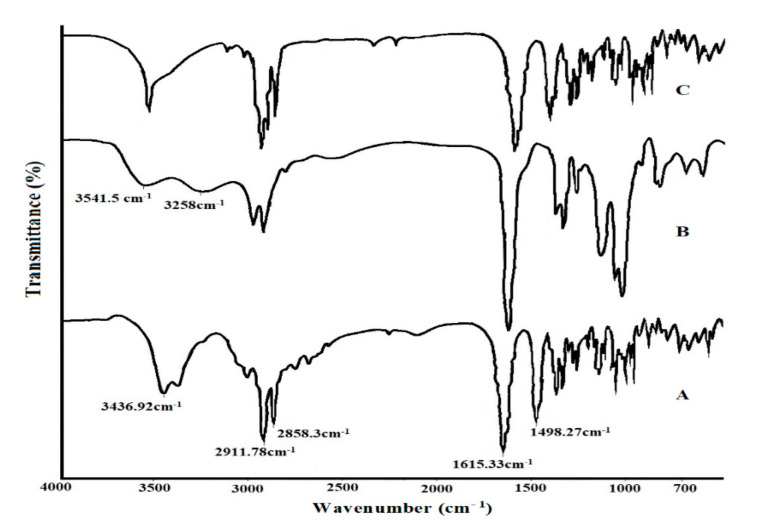
Overlay FTIR spectra of (A) pure Saxagliptin, (B) Eudragit RS100, and (C) optimized SLN formulation.

The overlay FTIR spectra of pure drug, Eudragit RS100, and optimized SLN formulation is shown in Figure 6. The spectrum of pure drug exhibited characteristic absorption peaks at 3436.92 cm^−1^ (corresponding to N-H stretch of secondary amine), 2911.78 cm^−1^, 2858.3 cm^−1^ (C-H stretching vibrations), 1615.33 cm^−1^, 1498.27 cm^−1^ (C-N stretching vibration), and intense peaks were observed between 1400 and 600 cm^−1^ representing the presence of an aromatic ring. The spectrum of Eudragit RS100 revealed characteristic peaks at 3541.5 cm^−1^ and 3258 cm^−1^ (CH aliphatic stretching). FT-IR spectrum of formulation depicted all the characteristic peaks corresponding to the drug confirming that there was no chemical interaction between the drug and the excipients used for SLNs preparation.

### 2.10. DSC Analysis

**Figure 7:**
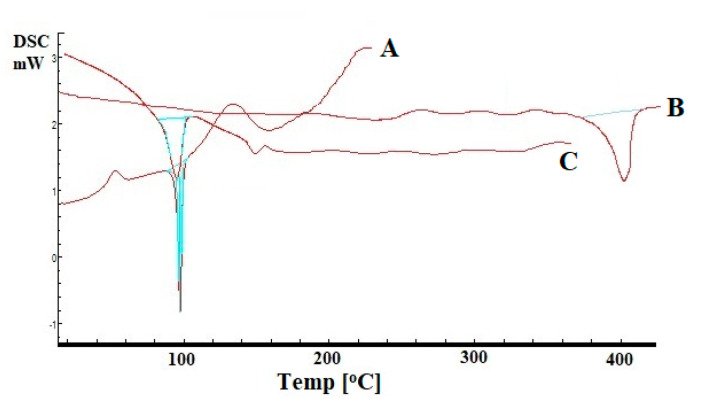
Overlay DSC thermograms of (A) pure Saxagliptin, (B) Eudragit RS100, and (C) optimized SLN formulation.

The recorded overlay DSC thermograms of pure drug, Eudragit RS100, and optimized SLN formulation are depicted in Figure 7. A characteristic sharp endothermic peak corresponding to melting point of Saxagliptin was observed at 97.56 °C (T_Onset_- 84.35 °C, T_Peak_- 97.56 °C, T_Endset_- 105.07 °C). DSC thermogram of Eudragit RS100 exhibited melting endotherm at 394.31 °C, which reflected that the melting point of the used polymer was variedly high from melting points of pure drug and drug in SLN formulation. Thermogram of optimized SLN formulation exhibited the endothermic peak corresponding to Saxagliptin melting, suggesting absence of any interaction and compatibility between drug and used excipients. The intensity of the endothermic peak corresponding to Saxagliptin was comparatively low, which can be corroborated to the dilution effect due to the presence of polymer.

### 2.11. Scanning Electron Microscopy (SEM)

**Figure 8:**
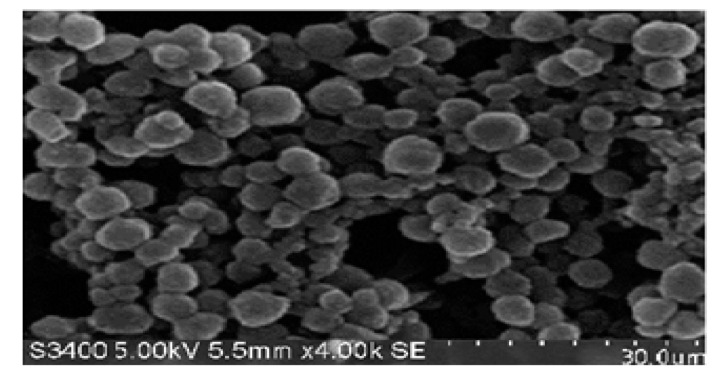
SEM image of the optimized SLN formulation.

SEM images captured for the optimized SLN formulation revealed roughly spherical irregular shaped Saxagliptin loaded SLNs (Figure 8). This noted morphological characters can be attributed to electrostatic interactions between anionic groups of lipid and surfactant. Moreover, not much free drug particles were observed onto the surface of SLNs, suggesting good encapsulation of the Saxagliptin.

### 2.12. In Vivo Studies

For estimation of Saxagliptin in plasma, plasma-extracted clear supernatant liquid aliquot was injected into HPLC system (specified in methodology section), analyzed, and processed. For the developed bioanalytical method, the noted linearity range was 5 to 15 mcg/mL with a quite good regression coefficient (r^2^ = 0.999; *n* = 6). The observed drug retention time was 5.46 min. The linearity between the analyte concentration and the peak area was established based on the findings of repeated runs. The developed bioanalytical method was very specific and robust. The method accuracy varied from 99.16% to 101.95% for intraday, and 96.08% to 103.12% for inter day operation at low (5 mcg/mL), moderate (10 mcg/mL), and higher (15 mcg/mL) drug concentrations.

Compared with the pure Saxagliptin, the pharmacokinetic analysis of optimized SLN formulation indicated considerable improvements in Cmax, Tmax, and bioavailability of the drug (Table 4). Moreover, in comparison to the pure drug, the SLN formulation depicted higher drug plasma levels. Saxagliptin’s pharmacokinetic data showed a C_max_ of 4.7 ± 0.16 mcg/mL and a T_max_ of 2 h. Whereas the optimized formulation exhibited a C_max_ of 5.1 ± 0.22 mcg/mL and a T_max_ of 3.0 ± 0.41 h. Using the below formula, relative bioavailability of pure drug and SLN formulation was calculated.

$$\mathrm{Relative}\;\mathrm{bioavailability}\;\left(\%\right)=\frac{\mathrm{AUC}\;\mathrm{of}\;\mathrm{SLN}\;\mathrm{formulation}}{\mathrm{AUC}\;\mathrm{of}\;\mathrm{drug}}\times \frac{\mathrm{Dose}\;\mathrm{of}\;\mathrm{drug}}{\mathrm{Dose}\;\mathrm{of}\;\mathrm{SLN}\;\mathrm{formulation}}$$

**Figure 9:**
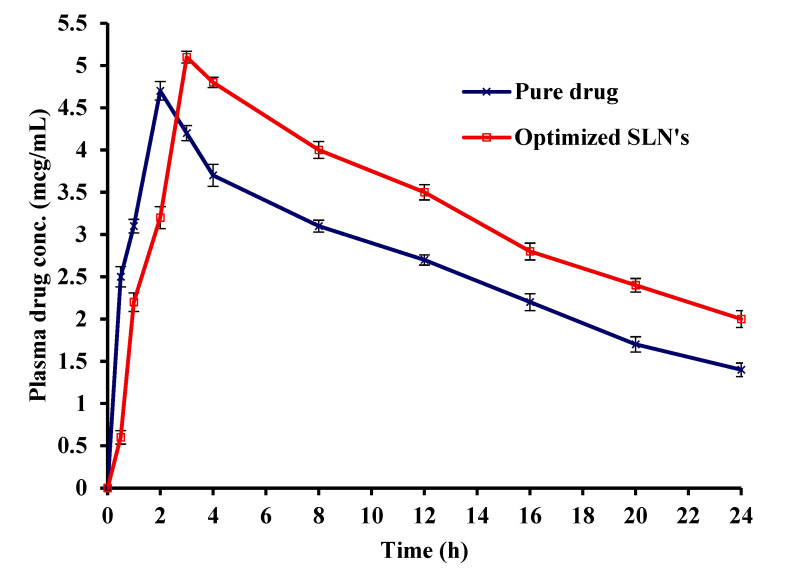
Plasma drug level profile of pure drug and optimizes SLN’s formulation.

The obtained data showed that the SLN formulation’s C_max_ was higher than that of the pure drug, indicating an increase in AUC and enhanced bioavailability. Increased AUC was seen as a result of bypassing the hepatic first-pass metabolism by targeting the drug in the gut. The increased T_max_ for the drug from 2 h to 3 ± 0.41 h can be corroborated to slow and prolonged drug release from SLN formulation (Figure 9). The enhanced bioavailability was accomplished by delivering the drug to the small intestine that contains the DPP-4 enzyme, which improves drug absorption and thereby bioavailability. The in vivo pharmacokinetics of produced Saxagliptin SLNs were consistent with those of SLNs performance in vitro. Both the investigations established that the presence of lipids and the Eudragit RS100 polymer coating were efficient in lowering drug release in areas other than the intestine and in enhancing Saxagliptin bioavailability.

### 2.13. Stability Studies

For assessing the stability of the prepared optimized batch and to know the shelf life and decomposition rate of the same, stability studies were conducted for the optimized Saxagliptin SLNs at elevated temperature and relative humidity (RH) storage conditions. As per the ICH guidelines, the optimized SLNs were placed in the stability chambers at specified temperature and relative humidity conditions, i.e., at 25 ± 2 °C/60 ± 5% RH (for Long Term Stability study), at 30 ± 2 °C/65 ± 5% RH (for Intermediate Term Stability study), and at 40 ± 2 °C/75 ± 5% RH (for Accelerated Stability study) [28]. The study period was 6 months during which samples were taken out from the stability chambers at predetermined intervals and assessed for any change in physical appearance (such as color, aggregation/clumping, melting, shape deformation, and particle size) and drug content. The noted results established that optimized SLNs were stable over the study period of 6 months (Table 5).

## 3. Materials and Methods

### 3.1. Materials

Saxagliptin was generously gifted by Hetero Drugs Ltd., Hyderabad, India. Eudragit RS100 was procured from Evonik, Rohm GmbH, Germany. Loba Chemie, Mumbai, India, provided the stearic acid. Poloxamer 188 and polyvinyl alcohol (PVA) were procured from BASF, Ludwigshafen, Germany, and HiMedia Laboratories Pvt. Ltd., Mumbai, India, respectively. Simulated intestinal fluid (SIF, pH 7.4) was procured from Biorelevant, London, UK. The rest of the chemical compounds, reagents, and solvents used were of analytical grade.

### 3.2. Solubility Studies

Saxagliptin solubility studies were carried out in two different lipids such as glyceryl monostearate and stearic acid. The individual lipids (5–10 mg) were taken into two beakers and were liquified by gentle heating up to 75 °C (which is ideally 5 °C higher than the melting temperatures of the lipids) using a water bath (Precision^®^, Thermo Scientific, New York, NY, USA). To the above, melted lipids of 5 mg of drug was added and was magnetically stirred for attaining uniform dispersion. Further, incremental amounts of respective lipids were added in portions under continuous heating and stirring until a clear solution was formed. The total amount of lipid added to obtain a clear solution was recorded and the formed saturated lipid solution was then dissolved in methanol, filtered, and the drug content was estimated using a simple, specific, and robust UV spectrophotometric method [29].

### 3.3. Experimental Design and Statistical Analysis

In the present work, optimization of critical material attributes and critical process parameters were performed using Design Expert Version-11 Software (Stat-Ease Inc., Minneapolis, MN, USA). CCD was implied as a response surface methodology approach where 4 independent variables were taken at 3 levels (−1, 0, +1) as low, medium, and high levels. Independent variables were A1: lipid amount, A2: polymer amount, A3: concentration of surfactant, and A4: speed of homogenization. The dependent variables were R1: size of particles and R2: entrapment efficiency. The response surface approach was used to predict and understand the interaction between 4 different factors and their influence on the dependent variables [30].

### 3.4. Preparation of Saxagliptin Solid Lipid Nanoparticles (SLNs)

The solvent injection technique was used at a temperature above the melting point of used lipids for preparing the SLNs. The weighed amount of stearic acid was melted (at 90 °C) and dissolved in ethanol using a water bath to obtain the lipid organic phase in which the drug (5 mg) was dispersed and sonicated (Sonics VCX-750 Vibra Cell Ultrasonic processor, Sonics and Materials Inc., Newtown, CT, USA) for 1 min. A total of 20 mL of aqueous phase containing surfactant was prepared and to this aqueous phase, lipid organic phase was added dropwise using an injection with continuous stirring at 1000 rpm for 1 h. Thereafter, the dispersion was homogenized (Yellow Line Ost Basic Company, Staufen, Germany) for 30 min followed by ultracentrifugation for 20 min at 15,000 rpm (Optima XPN-100 Ultracentrifuge, Beckman Coulter Inc., Brea, CA, USA). The obtained aggregates were dispersed in 10 mL of aqueous phase containing stabilizer. The dispersion was then stirred for 3 h at 1000 rpm and the formation of precipitates was allowed. The precipitates obtained were then dispersed in 10 mL of phosphate buffer (pH 6.8) containing dissolved Eudragit RS100 and sonicated for 2 min. The amount of Eudragit RS100 coating polymer used was in the weight ratio of 1:2 of total lipid to polymer to obtain enteric coated SLNs. Further, for precipitating the Eudragit RS100, acidic solution (pH 2) was added to the suspensions. Lastly, the obtained final suspensions were centrifuged for 30 min at 15,000 rpm (Thermo Scientific, Fiberlite™ H3-LV Large Volume Swinging Bucket Rotor, New York, NY, USA) and the Eudragit-coated SLNs were freeze-dried (Lyotrap-Plus, LTE Scientific Ltd., Oldham, UK) [31].

### 3.5. Determination of Particle Size, Polydispersity Index (PDI) and Zeta Potential

For determination of particle size, PDI, and Zeta potential, SLN samples were sufficiently diluted using water as a dispersant and analyzed using a dynamic light scattering-based particle size analyzer (Zetasizer Nano ZS Ultra, Malvern Instruments, Malvern, UK) [32].

### 3.6. Scanning Electron Microscopy (SEM)

The morphology and surface topography of developed SLNs was studied using cryo field emission gun scanning electron microscopy (cryo FEG-SEM, JSM-7600F, JEOL, Tokyo, Japan) [33].

### 3.7. Determination of Drug Entrapment Efficiency (EE) and Drug Loading (DL)

The drug entrapment efficiency and drug loading of the prepared SLNs were determined by dissolving a weighed quantity of SLNs in 20 mL of methanol via sonication (Sonics VCX-750 Vibra Cell Ultrasonic processor, Sonics and Materials Inc., USA). The resultant solution was then subjected to centrifugation at 10,000 rpm for 45 min (Thermo Scientific, Fiberlite™ H3-LV Large Volume Swinging Bucket Rotor, New York, USA). Later, the supernatant was separated, filtered through a 0.22 µ membrane filter (Millipore^®^, Merck, Darmstadt, Germany) and the free drug content in it was estimated using reversed phase HPLC (LC-4000, Jasco, Tokyo, Japan) equipped with a photodiode array (PDA) detector (MD-4015, Jasco, Tokyo, Japan), auto sampler (AS-4150, Jasco, Tokyo, Japan), and column oven (CO-4060, Jasco, Tokyo, Japan). Chromatographic separation was achieved using a Grace™ RP18-C18 column (250 mm × 4.6 mm, 5 µ, Fisher Scientific, Leicestershire, UK). The mobile phase contained 80% methanol and 20% water, which was filtered through the 0.22 µ membrane filter (Millipore^®^, Merck, Darmstadt, Germany) and degassed before analysis. The aliquots of 20 µL injection volume were injected at mobile phase flow rate of 0.8 mL/min and the column effluent was monitored at 212 nm. The run time for the Saxagliptin estimation was 7.15 min with a retention time of 4.19 min at 25 °C (Supplementary Data Figures S1 and S2). The chromatographic data were recorded, analyzed, and saved using Chromatography Data System software (ChromNAV 2.0, Jasco, Tokyo, Japan) [34]. After drug amount estimation, the EE and DL was calculated using the following formulas [35,36]:

$$\mathrm{Entrapment}\;\mathrm{efficiency}\;\left(\%\right)=\frac{\begin{array}{c} \mathrm{Amount}\;\mathrm{of}\;\mathrm{drug}\;\mathrm{in}\;\mathrm{SLN}-\\ \mathrm{amount}\;\mathrm{of}\;\mathrm{drug}\;\mathrm{in}\;\mathrm{supernatant}\;\mathrm{layer} \end{array}}{\mathrm{Amount}\;\mathrm{of}\;\mathrm{drug}\;\mathrm{in}\;\mathrm{SLN}}\times 100$$

(1) $$\mathrm{Drug}\;\mathrm{loading}\;\left(\%\right)=\frac{\begin{array}{c} \mathrm{Amount}\;\mathrm{of}\;\mathrm{drug}\;\mathrm{in}\;\mathrm{SLN}-\\ \;\mathrm{amount}\;\mathrm{of}\;\mathrm{drug}\;\mathrm{in}\;\mathrm{supernatant}\;\mathrm{layer} \end{array}}{\mathrm{Total}\;\mathrm{lipid}\;\mathrm{and}\;\mathrm{polymer}\;\mathrm{amount}}\times 100$$

### 3.8. In Vitro Diffusion Study

In vitro diffusion studies were performed using a dialysis bag, and the drug release from prepared SLNs was calculated. Briefly, 50 mg of drug loaded SLNs were placed inside the overnight soaked dialysis bag (HiMedia Laboratories Pvt. Ltd., Mumbai, India) which was dipped in 500 mL of dissolution medium (SIF pH 7.4), maintained at 37 ± 0.5 °C temperature, along with continuous stirring at 50 rpm for 24 h using a magnetic stirrer (Remi, MLV, Mumbai, India). At prefixed time intervals (0, 1, 2, 4, 8, 12, 16, 20, and 24 h), aliquots of 2 mL of dissolution media were withdrawn and replaced with equal quantity of fresh media. The HPLC technique was used to quantify the amount of drug released. Samples were directly injected into HPLC system and detected at 212 nm following the aforementioned operation parameters [34,37,38].

### 3.9. Fourier-Transform Infrared Spectroscopy (FTIR)

For evaluating the interactions and compatibility among drug excipients, FTIR studies were carried out using a FTIR spectrophotometer (Magna IR 750 series II, Nicolet, Madison, WI, USA) by pelleting the drug, excipients, and optimized SLNs with IR grade KBr in the ratio of 1:100 at 15,000 lb of pressure. The pellets were scanned in an inert atmosphere within the wave number range of 4000 cm^−1^ to 400 cm^−1^ [39].

### 3.10. Differential Scanning Calorimetry (DSC)

Thermal analysis of pure drug, excipients, and optimized SLN formulation were conducted using a differential scanning calorimeter (TA-60, Shimadzu, Kyoto, Japan) for evaluating the interactions and compatibility among the used drug excipients [40].

### 3.11. In Vivo Studies

The in vivo studies were performed at MNR College of Pharmacy, Hyderabad, India (proposal number: 1434/PO/Re/S/11/CPCSEA). Adult male albino Wistar rats were obtained from the animal housing facility, Department of Pharmacology, MNR College of Pharmacy, Hyderabad (an approved and registered facility under CPCSEA 2010). Animals were divided in 2 groups with 12 rats in each group. Group A was administered with pure Saxagliptin and Group B was administered with prepared and optimized SLNs loaded with Saxagliptin. Before starting the actual experiment, all animals were left fasting overnight with provision of only water. Pure drug and prepared SLNs at 1 mg/kg body weight dose were filled in capsule shells and provided to the respective group of animals orally. Blood samples were collected at specific time points (0, 2, 4, 8, 12, and 24 h) and were stored in microcentrifuge tubes containing heparin sodium (as anticoagulant). Further, plasma was harvested from these samples by centrifuging for 10 min at 4 °C and 10,000 rpm, and were stored at −20 °C in deep freeze until further analysis. The developed and validated HPLC method was used to analyze the plasma samples. A simple protein precipitation process using ethanol was implied to extract analytes from the plasma, and the separated clear supernatant layer was injected into the HPLC system for drug analysis [34].

### 3.12. Stability Studies

The drug may degrade during storage period which can be due to product instability or chemical degradation. To check any such sort of drug degradation in our optimized SLN formulation, stability studies were carried out over six months at accelerated and ambient conditions following ICH guidelines. At predetermined time intervals (0 day, 1 month, 3 months, and 6 months) samples were taken out from the stability chambers (Stability Chamber, Thermo Scientific, New York, NY, USA) and evaluated for their physical appearance and drug content [41].

## 4. Conclusions

In the present research vocation, a novel drug delivery system of Saxagliptin in the form of SLNs was designed, developed, and evaluated in great detail. For preparation of SLNs, a modified solvent injection method was adopted with implication of CCD as a Quality by Design (QbD) approach. The design outcomes indicated that independent variables (A1: amount of lipid, A2: amount of polymer, A3: surfactant concentration, and A4: homogenization speed) had significant effects on dependent variables particle size (R1) and entrapment efficiency (R2). Using 2FI model design, optimized Saxagliptin SLN formulation was prepared. In the in vivo pharmacokinetic study, the prepared and optimized SLNs exhibited enhanced bioavailability of Saxagliptin in comparison with the pure Saxagliptin. Thus, overall results suggested that the prepared Saxagliptin SLNs can be a promising alternative to conventional dosage forms for attaining enhanced bioavailability and efficacy in Type 2 antidiabetic therapy.

## Figures and Tables

**Table 1:** Experimental design and responses obtained for the prepared Saxagliptin SLNs.

Run	Independent Factors				Responses	
	A1 * (%)	A2 * (mg)	A3 * (mg)	A4 * (rpm)	R1 * (nm)	R2 * (%)
1.	10	40	3	4000	305	56.29
2.	15	20	2	3000	369	59.12
3.	10	60	2	4000	293	55.64
4.	5	60	2	4000	227	51.81
5.	15	60	2	5000	401	62.86
6.	10	60	2	3000	274	55.3
7.	10	20	1	3000	243	53.19
8.	5	40	2	5000	240	52.89
9.	10	60	3	3000	221	51.42
10.	10	60	1	5000	212	50.23
11.	5	40	1	4000	224	51.56
12.	5	40	2	3000	220	50.99
13.	15	60	3	4000	399	62.08
14.	5	60	1	3000	265	54.39
15.	15	40	1	3000	409	65.18
16.	15	60	2	3000	408	64.93
17.	15	40	1	5000	392	61.29
18.	10	20	2	4000	248	53.26
19.	5	20	3	3000	208	49.96
20.	10	20	3	5000	270	55.02
21.	5	60	1	5000	239	52.16
22.	5	60	3	4000	245	53.26
23.	15	60	1	3000	403	63.99
24.	15	40	2	4000	385	60.21
25.	10	40	1	4000	356	57.85
26.	5	40	3	5000	323	56.76
27.	10	40	2	5000	240	52.65
28.	5	20	2	5000	215	50.59
29.	15	20	1	5000	359	58.01
30.	5	20	1	4000	261	54.19
31.	10	40	1	3000	278	55.49
32.	10	60	1	4000	243	53.15
33.	15	20	3	4000	346	57.05
34.	15	60	1	4000	442	69.21
35.	15	60	3	5000	413	67.08
36.	15	40	3	3000	396	61.32
37.	15	20	1	4000	382	60.15
38.	5	20	1	3000	254	53.87
39.	10	40	3	4000	305	56.29

* Mean ± SD, *n* = 3; A1: Amount of lipid; A2: Amount of polymer; A3: Surfactant concentration; A4: Homogenization speed; R1: Particle size; and R2: Entrapment efficiency.

**Table 2:** Particle size ANOVA Response for 2FI model for the prepared Saxagliptin SLNs.

Source	Sum of Squares	df	Mean Square	F-Value	*p*-Value	Remark
**Model**	164.06	32	5.13	5.62	0.0311	**Significant**
A-A1	129.80	2	64.90	71.09	0.0002	
B-A2	4.07	2	2.04	2.23	0.2031	
C-A3	1.50	2	0.7501	0.8217	0.4914	
D-A4	0.7704	2	0.3852	0.4219	0.6771	
AB	2.79	4	0.6987	0.7654	0.5906	
AC	0.6087	4	0.1522	0.1667	0.9464	
AD	3.49	4	0.8717	0.9548	0.5040	
BC	2.49	4	0.6235	0.6830	0.6333	
BD	0.2405	4	0.0601	0.0659	0.9896	
CD	4.86	4	1.22	1.33	0.3735	
**Residual**	4.56	5	0.9129			
**Cor Total**	168.60	37				

**Table 3:** Entrapment efficiency ANOVA response for 2FI model for the prepared Saxagliptin SLNs.

Source	Sum of Squares	df	Mean Square	F-Value	*p*-Value	Remark
**Model**	936.55	32	29.27	5.68	0.0304	**Significant**
A-A1	677.36	2	338.68	65.73	0.0003	
B-A2	49.76	2	24.88	4.83	0.0680	
C-A3	12.52	2	6.26	1.21	0.3716	
D-A4	2.14	2	1.07	0.2074	0.8193	
AB	38.48	4	9.62	1.87	0.2543	
AC	11.71	4	2.93	0.5684	0.6980	
AD	11.23	4	2.81	0.5447	0.7120	
BC	3.94	4	0.9851	0.1912	0.9330	
BD	2.53	4	0.6328	0.1228	0.9681	
CD	53.89	4	13.47	2.62	0.1601	
**Residual**	25.76	5	5.15			
**Cor Total**	962.32	37				

**Table 4:** Noted pharmacokinetic parameters for pure Saxagliptin and optimized SLN formulation.

Product	C_max_ (mcg/mL) *	T_max_ (h) *	K_el_ (h^−1^) *	(AUC)_0_^t^ (ng/mL × h) *
Pure drug	4.7 ± 0.16	2	0.061	78 ± 5.45
Optimized SLN’s formulation	5.1 ± 0.22	3	0.036	112 ± 3.98

* Mean ± SD, *n* = 12; C_max_: Peak concentration or maximum concentration; T_max_: Time to reach maximum concentration; K_el_: Elimination rate constant; AUC: Area under the curve.

**Table 5:** Stability study data of the optimized SLN formulation.

Storage Condition	Sampling Interval	Physical Appearance	Drug Content (%)
25 ± 2 °C/60 ± 5% RH	0	No change	100
	1	No change	99.54 ± 0.58
	3	No change	98.85 ± 0.98
	6	No change	97.12 ± 0.45
30 ± 2 °C/65 ± 5% RH	0	No change	100
	1	No change	99.28 ± 0.85
	3	No change	98.71 ± 0.68
	6	No change	97.25 ± 0.94
40 ± 2 °C/75 ± 5% RH	0	No change	100
	1	No change	99.06 ± 0.39
	3	No change	98.29 ± 0.23
	6	No change	97.01 ± 0.85

## Data Availability

Not applicable.

## Supplementary Materials

The following supporting information can be downloaded at: https://www.mdpi.com/article/10.3390/molecules27217510/s1, Figure S1: RP-HPLC chromatogram for blank run; Figure S2: RP-HPLC chromatogram for analyte containing Saxagliptin.

## Sample Availability

Samples of the compounds are not available from the authors.
