# Supplementary material for: Formulation, Characterization, and Evaluation of Eudragit-Coated Saxagliptin Nanoparticles Using 3 Factorial Design Modules
Source: Molecules. 2022 Nov 3;27(21):7510. doi: 10.3390/molecules27217510 (PMC9653823; doi:10.3390/molecules27217510)
Supplement: Supplementary file 1 [file molecules-27-07510-s001.zip › molecules-1848929-supplementary.pdf]

SUPPLEMENTARY DATA:

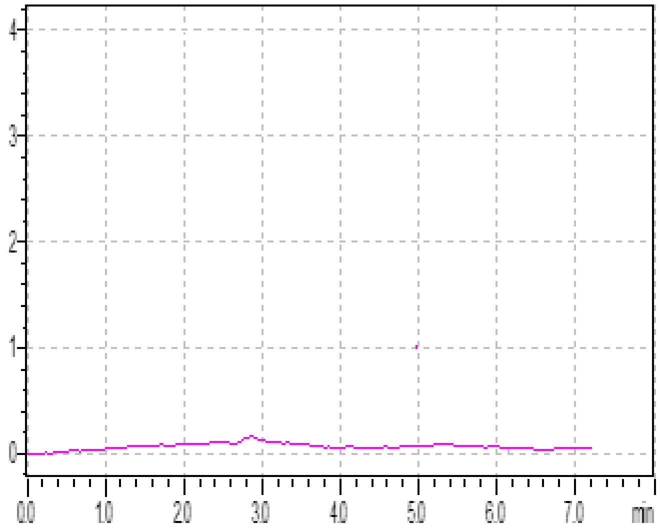

Figure S1: RP-HPLC chromatogram for blank run.

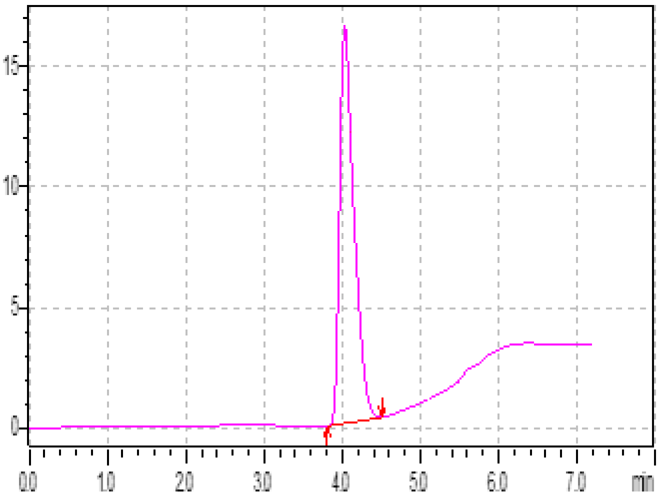

Figure S2: RP-HPLC chromatogram for analyte containing Saxagliptin.
